# Buske-Lowenstein Tumor: A Rare Cause of Genital Warts

**DOI:** 10.7759/cureus.23477

**Published:** 2022-03-25

**Authors:** Fernando Rivera-Alvarez, Bryan Kwon, Latha Ganti

**Affiliations:** 1 Emergency Medicine, University of Central Florida/HCA Healthcare Graduate Medical Education Consortium and Osceola Regional Medical Center, Kissimmee, USA; 2 Emergency Medicine, Brown University, Providence, USA; 3 Emergency Medicine, Envision Physician Services, Plantation, USA; 4 Emergency Medicine, University of Central Florida College of Medicine, Orlando, USA; 5 Emergency Medicine, Osceola Regional Medical Center, Kissimmee, USA; 6 Emergency Medicine, HCA Healthcare Graduate Medical Education Consortium Emergency Medicine Residency Program of Greater Orlando, Orlando, USA

**Keywords:** tumor, case report, condylomata acuminata, human papilloma virus, buschke-löwenstein

## Abstract

The Buschke-Löwenstein tumor (BLT), also known as giant condylomata acuminata (GCA), is a pseudo-epithelial proliferation engendered by the human papillomavirus (HPV). Interestingly, its location at the anal margin, or perianal skin, is rare. The authors present the case of a gentleman who became unstable while standing, stating that his ears were ringing. His emergency presentation, clinical course, and imaging findings are discussed. The patient presented with signs of condyloma acuminata and BLT. This can be excised through surgery and removed with the help of adjuvant treatments, but there is still much to learn about this disease.

## Introduction

First described by Buschke and Löwenstein in 1925, the Buschke-Löwenstein tumor (BLT) is a sexually transmitted infection that is primarily caused by the human papillomavirus (HPV). This infectious disease is relatively rare but is often identifiable by a precedent case of condylomata acuminata. Because condylomata acuminata always predates the BLT, this infectious disease is preventable with early diagnosis, hygiene, and wider control of sexually transmitted infections. The BLT can affect both sexes; however, it is most commonly found in males [1]. It often occurs in males between the ages of 40 and 60, which is represented in our case.

## Case presentation

This is the case of a 42-year-old male with no significant past medical history besides dyslipidemia and anal warts, reporting three months history of feeling off-balance. He also complained of ringing in both ears and reported worsening symptoms with standing. He denied syncope, chest pain, shortness of breath, headache, focal weakness, incoordination, double vision, melena, hematochezia, hematemesis, fever, chills, or neck pain. The patient also reported genital anal warts since age 19, and that area around warts had been oozing blood for some time. He was referred from his primary care physician for outpatient head computerized tomography (CT) scan, lab work, and cardiology consultation but symptoms worsened, and he came for evaluation. 

**Figure 1:**
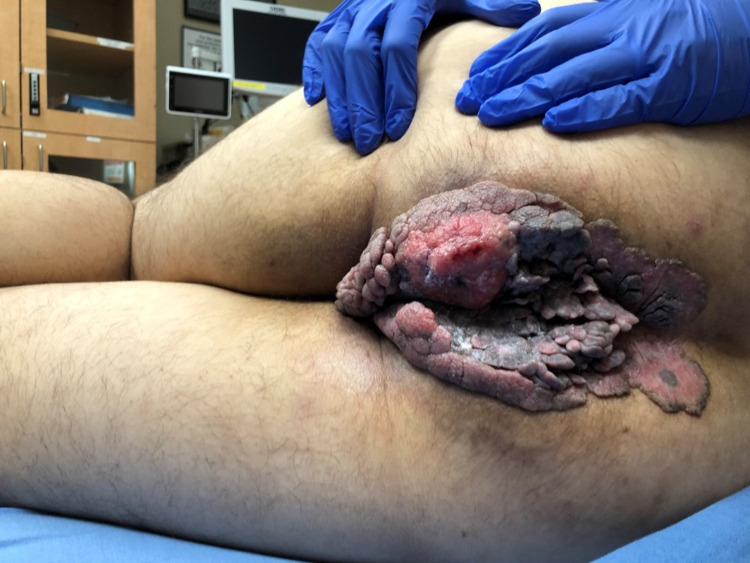
Condyloma acuminata with signs of Buschke-Löwenstein tumor.

The patient’s vital signs were within normal limits. Electrocardiogram revealed normal sinus rhythm. Physical examination was significant for mildly decreased breath sounds. Rectal examination showed a patent anus. CT of the head showed no abnormal acute findings. Laboratory evaluation revealed hemoglobin 6.1, white blood cell count 8.26, mean corpuscular volume 68.2, red cell distribution width (RDW) 15.6. The metabolic profile was without significant findings and was troponin negative. Human immunodeficiency virus test negative. The urinalysis was unremarkable. The patient was transfused with two units of packed red blood cells and admitted with gastroenterology consultation. The patient had surgery and biopsy with results consistent with condyloma acuminata with features of BLT and areas suspicious for invasive squamous cell carcinoma (Figure 1).

## Discussion

BLT is a rare condition, and its pathogenesis and natural history are not well understood. Tumors have a high proliferation rate and increased risk of transformation to malignancy. Our patient also had a p16 immunohistochemical stain which was reported as negative. The diagnosis remained unchanged.

The BLT clinically appears as a larger mass with budding similar to the form of a cauliflower. The excessive size poses danger to nearby organs and structures, as it can extend beyond tens of centimeters [2]. In our case, the tumor exhibits locoregional aggressiveness and a high relapse rate, reaching up to 66% even after excision [3].

Even though the incidence of the BLT has somewhat increased for the past 10 years, physicians still have a difficult time diagnosing the disease due to its similarities with ordinary condylomas or well-differentiated squamous cell carcinomas [4]. Lesions often present similarly; although the BLT can be recognized by its thick stratum corneum, an inclination for deep invasion, particularly in surrounding tissues, and pronounced papillary proliferation [3]. Due to the high recurrence rates of approximately 70% and inconsistent management across different levels of severity of this disease, they are problematic in controlling and treating.

Due to its rare nature, physicians do not have a general consensus concerning the treatment of the BLT. Even though there has been potential for several therapies, surgery is designated as the gold standard of treatment [3,5]. Unfortunately, the rate of relapse is upwards of 60% [2].

Several problems may appear after treatment due to reappearance or reinfection at the site of surgery. The high recurrence rate can prove to be an issue in regard to surgical treatment. Patients with cases presenting involvement of the bones or pelvis may require abdominoperineal resection. Additionally, complications can occur secondary to contamination of the wound in the perianal area by feces [4].

## Conclusions

Our patient presented with the commonly reported symptoms associated with condyloma acuminata and BLT. There is still much to learn about BLT in regard to treatment because of its rarity. Surgical excision with plastic reconstruction of skin defects appears to be one of the more promising options. Adjuvant treatments such as chemo- and radiotherapy may also have a role.
